# Two prophylactic medication approaches in addition to a pain control regimen for early medical abortion < 63 days’ gestation with mifepristone and misoprostol: study protocol for a randomized, controlled trial

**DOI:** 10.1186/s12978-016-0246-5

**Published:** 2016-10-12

**Authors:** Monica V. Dragoman, Daniel Grossman, Nathalie Kapp, Nguyen My Huong, Ndema Habib, Duong Lan Dung, Anand Tamang

**Affiliations:** 1 Department of Reproductive Health and Research, WHO, UNFP/UNDP/UNICEF/WHO/World Bank Special Programme of Research, Development and Research Training in Human Reproduction (HRP), Geneva, Switzerland; 2 Advancing New Standards in Reproductive Health (ANSIRH), Bixby Center for Global Reproductive Health, Department of Obstetrics, Gynecology and Reproductive Sciences, University of California, San Francisco, USA; 3 Ibis Reproductive Health, Oakland, CA USA; 4 National Hospital for Obstetrics and Gynecology, Hanoi, Viet Nam; 5 Center for Environment Health and Population Activities, Kathmandu, Nepal

**Keywords:** Medical abortion, Mifepristone, Misoprostol, Pain management

## Abstract

**Background:**

Pain is often cited as one of the worst features of medical abortion. Further, inadequate pain management may motivate some women to seek unnecessary clinical care. There is a need to identify effective methods for pain control in this setting.

**Methods/Design:**

We propose a randomized, placebo-controlled trial. 576 participants (288 nulliparous; 288 parous) from study sites in Nepal, South Africa and Vietnam will be randomly allocated to one of three treatments: (1) ibuprofen 400 mg PO and metoclopramide 10 mg PO; (2) tramadol 50 mg PO and a placebo; or (3) two placebo pills, to be taken immediately before misoprostol and repeated once four hours later. All women will be provided with supplementary analgesia for use as needed during the medical abortion. We hypothesize that women receiving prophylactic analgesia will report lower maximal pain scores in the first 8 h following misoprostol administration compared to women receiving placebos for medical abortion through 63 days’ gestation. Our primary objective is to determine whether prophylactic administration of ibuprofen and metoclopramide or tramadol provides superior pain relief compared to analgesia administration after pain begins, measured during the first eight hours after misoprostol administration. Secondary objectives include identifying covariates associated with higher reported pain scores; determining any impact of the study medicines on medical abortion success; and, qualitatively exploring women’s physical experiences of medical abortion, especially related to pain, and how can they be improved. Data sources include medical records, participant symptom diaries and interview data obtained on the day of enrollment, during the medical abortion, and at follow-up. Participants will be contacted via telephone on day 3 and return for follow-up will occur approximately 14 days after mifepristone, concluding study participation. A subset of 42 women will also be invited to undergo in-depth qualitative interviews following study completion.

**Discussion:**

Although pain is one of the most common side effects encountered with medical abortion, little is known about optimal pain management for this process. This multi-arm trial design offers an efficient approach to evaluating two prophylactic pain management regimens compared to use of pain medication as needed.

**Trial registration:**

ACTRN12613000017729 (Prospectively registered 8/1/2013).

## Plain English summary

Pain is a predictable feature of the medical abortion process, and for some women, pain may be intense. Although pain is the most common side effect encountered with medical abortion, little is known about optimal pain management during this process, and there is a clear need to identify effective methods for pain control. Our study is a randomized, placebo-controlled trial of different options for pain relief during early medical abortion with mifepristone and misoprostol. It is designed to determine whether initiating different medicines before pain begins (tramadol, a weak narcotic, or ibuprofen plus metoclopramide, an anti-nausea medicine) is superior to taking medicines only once pain begins during the medical abortion process.

## Background

Pain is a predictable feature of the medical abortion process, and for some women, pain may be intense [1, 2]. Pain is typically most acute following administration of prostaglandins or their analogues and most intense prior to pregnancy expulsion, typically occurring approximately 4 h following misoprostol administration for the combined regimen (mifepristone and misoprostol) for medical abortion [3–5]. As pain is often cited as one of the worst features of medical abortion [6], and given that inadequate pain management may motivate some women to seek unnecessary clinical care, there is a clear need to identify the most effective methods of pain control during the abortion process.

Current guidelines from the World Health Organization (WHO) recommend use of non-steroidal anti-inflammatory drugs (NSAIDs) during medical abortion for pain management; however, these recommendations draw from limited available evidence [7–11]. WHO notes that further research to determine best pain management options, including timing of medication administration, for medical abortion provided both before and after twelve weeks is a priority [12].

The NSAID Ibuprofen is commonly recommended for pain management with medical abortion. Raymond et al. demonstrated that prophylactic ibuprofen alone does not sufficiently address women’s pain with early medical abortion (EMA, generally defined as abortion up to 63 days from last menstrual period) [13]. However, we propose to investigate the effects of prophylactic administration of ibuprofen co-administered with metoclopramide because we hypothesize that the combination of these medicines may offer improved pain control as well as a practical approach to outpatient pain management that builds on current recommendations for the provision of NSAIDs to treat pain with EMA [12].

Nausea is reported by approximately half and vomiting is reported by one-third of all women undergoing medical abortion [14]. These symptoms are related both to the physiologic effects of early pregnancy as well as known side effects of the abortifacients, particularly misoprostol. Metoclopramide is an anti-emetic and pro-kinetic agent that is used commonly to treat a variety of gastrointestinal disorders, including nausea and vomiting associated with pregnancy [15]. It is rapidly and well-absorbed following oral administration with peak plasma concentrations achieved at 1–2 h after ingestion. Typically, it is prescribed for use every four to eight hours.

No studies have previously investigated the use of oral metoclopramide as part of a pain control regimen for EMA. However, several small randomized trials describe the use of adjuvant metoclopramide with intravenous patient-controlled analgesia (IV PCA) during second trimester medical abortion [16–18]. Women receiving IV metoclopramide 10 mg generally reported less nausea and vomiting and lower pain scores compared to controls. Though not statistically significant, it was also observed that women receiving metoclopramide tended toward shorter induction-to-delivery intervals (ranging from 3–7 h). It has been theorized that metoclopramide may help coordinate smooth muscle contraction of the uterus and fallopian tubes following exposure to prostaglandins, resulting in decreased discomfort and improved expulsive force [17]. We postulate that metoclopramide not only offers potential to improve the acceptability of medical abortion among some women by reducing side effects, but it may also expedite pregnancy expulsion and contribute to an overall decrease in pain when provided as adjuvant therapy to ibuprofen.

In addition, we propose to investigate whether prophylactic administration of tramadol may be superior to placebo in reducing women’s pain with EMA. Tramadol is a non-traditional opioid that is structurally similar to codeine and morphine; it is indicated for the use of acute and chronic moderate-to-severe pain. Unlike morphine and codeine, tramadol is a racemic mixture of both enantiomers, and they and their metabolites contribute to analgesia via different mechanisms. They bind centrally to μ-opioid receptors as well as inhibit norepinephrine and serotonin reuptake, resulting in inhibition of both transmission and perception of pain [19]. Following oral administration, tramadol is rapidly and almost completely absorbed with peak plasma concentrations achieved at 1.6–1.9 h. It also can be provided every four hours for pain.

The most common side effects associated with tramadol include nausea, dizziness, drowsiness and vomiting; these are reported at a low frequency among users, ranging between 2–8%, equivalent to the frequency of these side effects among users of other opioid medications [20]. However, tramadol is associated with a lower incidence of respiratory depression, constipation and abuse compared to morphine and codeine. Tramadol’s performance as a pain medication, safety profile and tolerability contribute to its recognition as an important pain medication for short-term and chronic pain management across a variety of indications. A number of international guidelines specifically recommend tramadol, not just weak opioids, as a choice medication for pain management [20–23].

Tramadol, provided intravenously and intramuscularly, has been described as an effective agent to treat labor pain as well as pain associated with medical abortion in the second trimester [18, 24, 25]. In addition, women randomized to receive a tramadol rectal suppository prior to surgical abortion required less intraoperative anesthesia and rescue analgesia than women who received indomethacin (an NSAID); they also reported lower postoperative pain scores [26]. A small number of multiparous women undergoing IUD insertion were randomized to oral tramadol, naproxen (an NSAID) or placebo one hour prior to their procedure; women who received tramadol reported the lowest pain scores [27]. This limited evidence demonstrates that tramadol may be an effective agent to treat pain associated with uterine cramping, perhaps superior to NSAIDs, and suggests a role to address pain from EMA.

Evaluation of new pain management strategies to prevent or minimize pain accompanying medical abortion is important for improving the quality of medical abortion services for women. A major challenge in evaluating new treatments for clinical care is the considerable time and resources needed for conducting randomized, controlled trials. Given the evidence regarding prophylactic ibuprofen administration alone and paucity of data informing pain management with EMA overall, a multi-arm trial design offers an efficient approach to evaluating two experimental treatments compared to a placebo control arm rather than conducting multiple RCTs [28].

The primary objective of this study is to determine whether prophylactic administration of ibuprofen and metoclopramide or tramadol and placebo are superior to placebo alone combined with analgesia administration after pain begins during the medical abortion process, measured in the first eight hours following misoprostol administration. Secondary objectives include identifying covariates other than the pain management regimen that may be associated with higher reported pain levels. Also, we will report if prophylactic use of these medications impacts the effectiveness of the medical abortion regimen. Finally, among a subset of participants, we will qualitatively explore women’s physical experiences of medical abortion, especially related to pain, and share insights into how they might be improved from women’s perspectives.

## Methods

### Study design

**Fig. 1:**
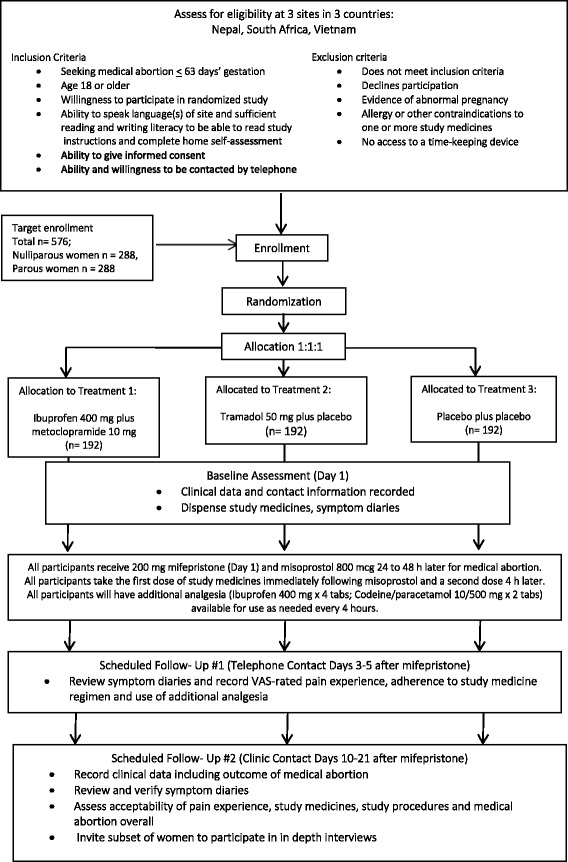
CONSORT/Spirit Flow Diagram

We propose a multi-center, three-arm, blinded, randomized, controlled trial; participants will be randomized in a 1:1:1 allocation ratio to either one of two active prophylactic pain management arms or a placebo arm while undergoing early medical abortion (≤63 days from last menstrual period) with mifepristone and misoprostol. All participants will also have access to additional analgesia to use as needed at their own discretion during the medical abortion process (See Fig. 1).

When a woman presents requesting EMA, she will be screened for inclusion and exclusion criteria; only eligible women who provide their informed and voluntary consent will be recruited to the trial. Consent for the abortion procedure will be obtained separate from the consent to participate in research.

Participants will ingest mifepristone 200 mg orally at the study site on day one. Approximately 24–48 h after taking mifepristone, women will take the first dose of study medicines at home immediately prior to use of 800 mcg misoprostol, by vaginal (Nepal, Vietnam) or sublingual (South Africa, Vietnam), routes according to the standard practice at the site. The second dose of study medicines will be taken four hours later. Women will complete a diary to note the times the medications were taken and pain levels recorded on a 0-10 scale. Women will be contacted via telephone between days three to five after taking mifepristone. This telephone contact, lasting approximately ten minutes, will review the maximum level of pain participants experienced during the first 8 and 24 h after misoprostol, their adherence to the study medicine regimen and use of additional analgesia. Women will also be asked to attend clinic for a scheduled follow-up appointment approximately two weeks after initiating the medical abortion process. At this visit, an assessment for completion of the medical abortion will be performed, the diary will be reviewed, and additional data will be collected regarding acceptability of the medical abortion overall, the pain medication regimens, and the pain experience.

### Setting and participants

Women will be recruited from sites where safe and legal medical abortion services are well-established in diverse and regionally representative low resource countries. Specifically, the Paropkar Maternity and Women’s Hospital in Kathmandu, Nepal, Job Tabane Hospital in Rustenburg, South Africa, and the National Hospital of Obstetrics and Gynecology in Hanoi, Vietnam, are hosting this study.

All participants will be administered mifepristone in clinic to initiate the medical abortion and will be instructed to use misoprostol and the study medicines at home according to standard practice. In Nepal and Vietnam, women also have the option to use misoprostol (and for the purposes of this trial, the study medicines) under supervision in the clinic until the time of expulsion; typically, a minority of women undergoing medical abortion at these sites choose to complete the medical abortion in clinic.

Women ages 18 years and older seeking medical abortion up to 63 days’ gestation at these sites, who report a willingness to participate in the study, are fluent in the language of site, possess sufficient literacy to adhere to study instructions and complete home self-assessments, and agree to be contacted by telephone are eligible for participation. Women will be excluded if any pregnancy abnormalities (e.g. multiple gestation, molar, ectopic, or non-viable pregnancy) or allergies/contraindications to use of mifepristone and misoprostol or any of the study medications are detected. Additionally, women who report no access to a time-keeping device or an inability to return to the study site with no option for telephone contact will be excluded.

### Intervention

Participants will be randomized to receive one of the following:

1. ibuprofen 400 mg PO and metoclopramide 10 mg PO;
2. tramadol 50 mg PO and a placebo; or
3. two placebo pills, to be taken immediately before misoprostol and repeated once four hours later.

All women will also be provided with additional analgesia (4 tablets of ibuprofen 400 mg and 2 tablets of codeine 10 mg/ paracetamol 500 mg) for use as needed during the medical abortion at their discretion. All women will receive detailed instructions about all study medicines, including the timing, route and indications for use of the various medications they are supplied.

The trial statistician at WHO centrally generated the randomization and treatment allocation scheme, based on computer-generated random permuted blocks (using STATA statistical software, version 10.0, College Station, TX) to share with the supervising trial pharmacist. Block sizes will be randomized and information on block sizes will be masked to all investigators during treatment allocation across study arms. Treatment allocation is stratified according to parity (sample will be 50% nulliparous and 50% parous in each treatment arm) in order to avoid imbalances across the three treatment groups. All study medicines are pre-packaged in opaque bags, labelled with subject numbers and sealed off-site in accord with the centrally-generated randomization and treatment allocation scheme, supervised by a skilled pharmacist, then transported for dispensing at research sites. Women will receive blind treatments from study staff at research sites by sequential assignment of the pre-packaged study medicines according to subject number.

### Study outcomes

Our primary outcome is maximum self-reported pain during the first 8 h after misoprostol administration and use of the first dose of study medicines. Pain will be measured by an 11-point numeric visual analogue scale (VAS); values range from 0–10 where 0 is equivalent to no pain and 10 is worst possible pain.

Secondary outcomes include:

- o Maximum pain as measured by VAS over first 24-h period
- o Use of any additional analgesic medication
- o Use of any supplemental narcotic
- o Effectiveness of the medical abortion regimen, defined as successful completion without additional surgical intervention.

### Sample size

The sample size is based on our primary outcome. VAS measurements for self-assessment of pain have been extensively validated in the literature [29]. Previous research found that the maximum VAS score with early medical abortion with mifepristone and misoprostol was 7, ranging from 5–8 [7–10]. Studies have also reported that the minimally clinically significant difference in VAS scores is between 1.5 and 2 [30, 31]. In order to detect a reduction in maximum VAS scores in the first 8 h from 7 to 5.5, assuming a standard deviation of 3.0, using a two-sided alpha of 0.05 and power of 90% while accounting for 10% loss to follow up, we will need 96 women in each study arm. We also want to determine the effect of prophylactic medication by parity among nulliparous and parous women. Due to stratification by parity, we plan to enroll a total of 576 women, 192 women per treatment arm, half of whom will be nulliparous. Because our treatment arms are independent of one another, and the aim being to assess independently the efficacy of ibuprofen and metoclopramide regimen or tramadol in reducing pain, we do not have to account for a multiplicity adjustment in our sample size calculations. A multiplicity adjustment for sample size is needed in multiple comparisons of interventions containing related regimens or in case of interventions with a dose-response relationship [28].

### Data collection

Data will be collected from participants at four time points:

1. At enrollment, women will be asked to provide basic demographic information and medical history, and clinical information following assessment by a health provider will be recorded.
2. Following initiation of the medical abortion process with mifepristone, women will be asked to record details about the timing and use of misoprostol and study medicines as well as any additional analgesia, self- assessments of pain and the experience of known side effects associated with medical abortion and the study medicines, such as nausea, vomiting or bleeding. As the majority of participants will be taking misoprostol and the study medicines at home, we are relying on women to record their own data. They will be provided a detailed orientation to the diary and the imperative for timely and complete data collection will be communicated prior to discharge from the clinic.
3. Women will be contacted via telephone on days three to five following mifepristone use (days 1–3 after misoprostol) to document women’s pain assessments during the first 8 and 24 h after misoprostol, use of study medicines and any additional analgesia.
4. Approximately two weeks following enrollment, women will be asked to attend clinic to determine abortion completion and to collect clinical data, verify and record data from their personal diaries and to inquire about the acceptability of the medical abortion process, study medicines, additional analgesia and study procedures overall.

Any discrepancies in data collected at the time of telephone contact, recorded in the participant diary or reported at follow up will be resolved at the time of follow up with the woman. Data collection forms and participant diaries will be prepared in English; these forms will be translated as necessary and translations verified prior to use in the respective countries. All required measures to protect participant confidentiality during data collection and data management will be implemented according to GCP standards.

### Data management

Guidelines were prepared for interviewing and collecting data, recording data on paper case report forms and entering these data in OpenClinica, a GCP-compliant, password-protected, web-based application for data entry and management. All data collectors, data entry operators and data managers at the sites will be trained before initiating the trial. Participating centers are requested to first record data on paper data collection forms and then double-enter data into the online OC system as soon as possible (minimally on a weekly basis). Queries for missing data, data errors and inconsistencies are automatically generated in the OC system and resolved accordingly by the local data manager with the assistance of the clinical trial manager at WHO. All participating sites will be subject to periodic on-site study monitoring visits for trial audits as part of our plan to assure quality; these visits will be lead by the trial sponsor and have oversight by the WHO clinical trial manager.

### Data analysis

Standard datasets will be generated from the final OpenClinica study database for analysis. A study participant flowchart will be prepared, as per CONSORT guidelines to present the numbers of women screened, randomized, allocated interventions, discontinued or lost to follow-up and analyzed [32]. The rate of follow-up of participants across study arms will be compared using the Kaplan-Meier survival method. Baseline characteristics, and results for primary and secondary outcomes for all randomized participants will be summarized according to treatment allocation. For categorical variables, the frequencies and crude percentages will be reported. Type and frequency of additional analgesia use and medical abortion effectiveness will be treated as categorical variables. For quantitative variables, crude mean estimates and standard errors will be provided for normally distributed variables; and medians, interquartile ranges, minima and maxima will be reported for variables with skewed distributions. Pain scores will be treated as continuous variables with comparison between study arms being done using parametric tests as long as normality assumptions hold. In this case, Analysis of Variance (ANOVA) will be applied to test for differences in pain score between the allocated arms, using both ITT and per-protocol (PP) study populations, A generalized linear model (GLM) using a normal distribution and an identity link will be used to compare pain levels between arms while adjusting for parity and country, randomization stratification factors. In the case of a skewed distribution of VAS pain scores, a Kruskal-Wallis non-parametric test will be used to crudely compare median pain scores between the three arms; in this case, transformation of the pain scores to a normal distribution may be considered prior to using the GLM model.

The GLM model will also be used to evaluate important covariates other than pain medication regimen associated with higher reported pain levels as long as normality assumptions hold. Two-sided tests and 5% significance levels will be used with 95% confidence intervals for all relevant parameters. The SAS statistical package will be used for the statistical analyses [33]. Open-ended questions will be listed and coded for meaningful comparisons of their distributions.

Log-binomial regression will be used to estimate and compare the risk of exposure to the study interventions on secondary outcomes, including use of any additional analgesic medication, use of any supplemental narcotic and effectiveness of the medical abortion regimen, while adjusting for randomization stratification factors.

### In-depth interviews

A subset of women (*n* = 42) will be invited to participate in an in-depth interview after completion of their medical abortion. Because young age and parity have been associated with pain during medical abortion, we plan to purposely recruit half the sample of 42 women for in-depth interviews to be nulliparous (and most will also be young). To try to capture a range of pain experiences, we will also purposively sample women who report different pain intensities associated with their medical abortion identified at the time of the follow-up interview. These interviews will be audio-recorded, transcribed verbatim, and coded using the qualitative software Atlas Ti. Two investigators will independently conduct a content analysis of the interview transcripts using the same software to identify emerging themes.

### Safety considerations

We recruited clinical pharmacists to conduct a systematic evaluation of the literature regarding any potential drug interactions among those proposed for use in the trial. Theoretically, the use of misoprostol, which is a prostaglandin analogue, with NSAIDs could result in a drug interaction which decreases the effectiveness of the medical abortion. Despite this theoretical concern, clinical studies have not demonstrated a difference [13, 34, 35]. Further, no known drug interactions between tramadol and mifepristone or misoprostol, metoclopramide and mifepristone or misoprostol or between ibuprofen and metoclopramide have been reported [36].

Metoclopramide has been linked to an increased risk of acute dystonic reactions, affecting 0.2% of users [37]. Tardive dyskinesia can manifest as involuntary and repetitive movements of the body, even after the drugs are no longer taken and can be irreversible (Package labeling for metoclopramide, TEVA pharmaceuticals USA). In our investigation, participants will have short-term (two doses) and low-dose exposure to this medication, mitigating the risk of this rare occurrence.

### Adverse events

Adverse events are defined as any untoward medical occurrence that a participant may experience during the course of the study, regardless of its relationship to study product use. We will record only new events, or a worsening of an existing condition, as AEs in the trial. In our study, the following routine trial measurements will not be considered AEs because they are defined as trial endpoints: abortion; and symptoms associated with medical abortion (e.g. pain, vaginal bleeding, fever, chills, nausea, vomiting and diarrhea).

A serious adverse event (SAE) is defined as any untoward medical occurrence that:

- Is life threatening or results in death.
- Requires in-patient hospitalization or prolongs existing hospitalization.
- Results in persistent or significant disability/incapacity.
- Is a congenital abnormality/birth defect (in the offspring of a participant).
- Jeopardizes participant and required medical/surgical intervention to prevent serious outcome, *or*
- Any other event that the investigator considers serious.

Details about each AE will be recorded on an Adverse Event Form, and about SAEs on a Serious Adverse Event Form. In accordance with ICH guidelines, these forms will require the following information to be collected: diagnosis, onset date, resolution date, source of information, severity, treatment given, relationship to study product, action taken, and comments. The forms will be completed by study staff and the Principal Investigator informed as soon as possible. All SAEs must be reported to the WHO project manager within 24 h of when the PI was notified; WHO will facilitate timely reporting to the required regulatory and advisory bodies providing oversight to the study. Clinical trial insurance has been secured to cover participants in the study at all collaborating sites.

### Data safety monitoring board

An independent data and safety monitoring board will be established to review efficacy and safety data. The DSMB will be made up of selected external individuals with relevant expertise in medical abortion, biostatistics, epidemiology and ethics. If the DSMB feels the data confers significant harm or benefit associated with one or more of the treatment arms and recommends discontinuation, recruitment to the treatment arm or the entire study may be stopped accordingly. Stopping rules will be based on Haybittle-Peto boundaries for efficacy estimates. One formal interim analyses of efficacy will be performed when 50% of the expected sample size have enrolled; no correction of the reported P value for these interim tests will be performed. The board will also consider safety data in making any decision to stop the study. The board may consult with outside experts before making any decision regarding study termination or continuation.

### Ethics

The trial is being conducted in accordance with the principles of Good Clinical Practice [38] Ethical approval for the study protocol has been granted both by the WHO Ethics Review Committee and country-specific review bodies including the Nepal Health Research Council and the Ethics Review Board of the Ministry of Health in Vietnam. It is currently undergoing review by the Human Research Ethics Committee of the University of Witwatersrand in Johannesburg, South Africa and Allendale Investigational Review Board in Old Lyme, Connecticut, USA; recruitment in South Africa will not begin until all approvals are obtained.

All participants will provide written consent before enrolling in the trial or commencing the follow-up interview. All client records, written, recorded and transcribed data will be de-identified and stored securely. No names of participants (or others mentioned) or locations will be used in the analysis or report writing. Confidentiality will be maintained by assigning coded identifiers to participant names (with a master list stored separately). Participants will be able to withdraw at any point should they no longer wish to remain in the study. Participants are provided remuneration for expenses related to clinic and telephone follow-up, but not for participation in the trial.

## Discussion

It is important to recognize that though the report of pain is common across settings where medical abortion is provided, there has been little investigation into optimal approaches to pain management outside of developed countries. Individual experiences of pain, responses to pain and responses to pain medication are complex and may differ according to ethnicity, socio-economic status, cultural factors, physiology and genetics [39]. To date, studies investigating ibuprofen to treat pain associated with EMA report on predominantly populations of women in the United States, Canada and Israel [9, 10, 13, 34]. In contrast to prior studies, our proposal emphasizes investigation of new pain management approaches in populations of women from Africa and Asia.

WHO currently recommends vaginal, buccal and sublingual routes for provision of misoprostol as part of a combined mifepristone and misoprostol regimen for medical abortion through 63 days’ gestation, and our partner sites offer various recommended routes according to their standard practice [12]. The safety and effectiveness of the combined regimen is maintained regardless of route of administration; however, there can be differences in the frequency of side effects, including pain, nausea, vomiting and diarrhea, due to variations in pharmacokinetics that will need to be accounted for during analysis and interpretation of our results [40]. Misoprostol provided sublingually is associated with the fastest onset of action, the shortest time to peak concentration (30 min), the highest peak concentration and greatest bioavailability in comparison to other routes. Both the buccal and vaginal misoprostol absorption curves are similar, achieving a peak concentration at around 60–80 min after administration, and the timing and intensity of their effects on uterine contractility are very similar. However, the drug levels attained by buccal administration are consistently lower for six hours compared to vaginal administration. The likelihood of side effects is greatest for sublingual and lowest for buccal administration.

Our main concerns regarding execution of this investigation center on participants’ adequate adherence to study procedures at home, including self-administration of study medication and completing the diary to record pain, timing of expulsion of pregnancy and symptoms associated with the medical abortion process. The study medications and additional analgesics will be packaged and color-coded with clear instructions for use to facilitate ease of home administration. During training of study staff, we will emphasize clear communication of instructions for medication use and diary entry; all participants will be encouraged to ask questions about issues that are unclear as well as to contact the study sites should concerns arise during the study. Importantly, we are planning to stage the roll out of the implementation of the study across countries. Following completion of the first 30 participants enrolled in the study at our first site, we will evaluate the feasibility of our study procedures and amend them, if necessary, to ensure quality data collection moving forward.

Additionally, we also hope to minimize participants’ loss to follow-up. Our partners have high-volume sites offering medical abortion and report that a high proportion of patients return for follow-up appointments to confirm completion of the abortion. We are instituting telephone contact on days 3 to 5 following mifepristone administration to collect data relevant to our primary objective. To encourage follow-up around day 14, women will have the option for clinic (preferred) or telephone contact. Should women not be able to physically return to the clinic for follow-up, we will be able to complete the follow-up questionnaires via telephone with participants. Women will also be offered remuneration for their time and travel associated with study procedures. These measures should attenuate significant loss to follow-up in our investigation. We have accounted for a 10% loss-to-follow-up rate in our sample size calculation; we do not expect to exceed this anticipated loss.

Both ibuprofen and metoclopramide are included on the WHO Essential Medicines List, globally available, and relatively inexpensive [41]. Given that both medicines are accessible and ibuprofen is already endorsed for use with EMA in international and national guidelines, implementation of routine use of this regimen would be fairly easy if any benefit to the pain experience was determined.

Ideally, a simple approach to pain management is preferable to a more complicated regimen requiring multiple medications. If tramadol offered benefit, advocating for its use with EMA would be easier to implement as a single agent versus a multi-agent regimen. Currently, tramadol is not included on the WHO Essential Medicines List or considered for routine use with EMA, but it is globally registered and generally available with prescription. With positive findings, use of tramadol in practice for this indication would likely be feasible, but might require additional advocacy to ensure availability.

## Abbreviations

ANOVA: Analysis of variance
CONSORT: Consolidated standards of reporting trials
DSMB: Data safety monitoring board
EMA: Early medical abortion
ITT: Intention-to-treat
IV PCA: Intravenous patient-controlled analgesia
NSAID: Nonsteroidal anti-inflammatory drug
OC: OpenClinica
RCT: Randomized, controlled trial
VAS: Visual analog scale
WHO: World health organization

## References

[1] Wiebe ER (1999). Comparing abortion induced with methotrexate and misoprostol to methotrexate alone. Contraception.

[2] Wiebe ER (1997). Choosing between surgical abortions and medical abortions induced with methotrexate and misoprostol. Contraception.

[3] Spitz IM, Bardin CW, Benton L, Robbins A (1998). Early pregnancy termination with mifepristone and misoprostol in the United States. NEJM.

[4] Winikoff B (1995). Acceptability of medical abortion in early pregnancy. Fam Plann Perspect.

[5] Fiala C, Gemzel-Danielsson K (2006). Review of medical abortion using mifepristone in combination with a prostaglandin analogue. Contraception.

[6] Winikoff B, Ellertson C, Elul B, Sivin I (1998). Acceptability and feasibility of early pregnancy termination by mifepristone-misoprostol. Results of a large multicenter trial in the United States. Mifepristone Clinical Trials Group. Arch Fam Med.

[7] Urquhart D, Templeton A, Shinewi F, Chapman M, Hawkins K, McGarry J (1997). The efficacy and tolerance of mifepristone and prostaglandin in termination of pregnancy of less than 63 days gestation; UK multicentre study—final results. Contraception.

[8] Fiala C, Swahn ML, Stephansson O, Gemzell-Danielsson K (2005). The effect of non-steroidal anti-inflammatory drugs on medical abortion with mifepristone and misoprostol at 13-22 weeks gestation. Human Reprod (Oxford, England).

[9] Wiebe E (2001). Pain control in medical abortion. Int J Gynaecol Obstet.

[10] Livshits A, Machtinger R, David LB, Spira M, Moshe-Zahav A, Seidman DS (2009). Ibuprofen and paracetamol for pain relief during medical abortion: a double-blind randomized controlled study. Fertil Steril.

[11] Jackson E, Kapp N (2011). Pain control in first-trimester and second-trimester medical termination of pregnancy: a systematic review. Contraception.

[12] World Health Organization (2012). Safe abortion: technical and policy guidance for health systems.

[13] Raymond EG, Weaver MA, Louie KS, Dean G, Porsch L, Lichtenberg ES (2013). Prophylactic compared with therapeutic ibuprofen analgesia in first-trimester medical abortion: a randomized controlled trial. Obstet Gynecol.

[14] Blumenthal P, Clark S, Coyaji K, Ellertson C, Fiala C, Mazibuko T, et al. Providing medical abortion in low-resource settings: an introductory guidebook. 2nd ed. New York: Gynuity Health Projects; 2009.

[15] Nausea and vomiting of pregnancy. ACOG practice bulletin. Obstet Gynecol. 2015;126(3):e12-24. 10.1097/AOG.0000000000001048 26287788

[16] Rosenblatt WH, Cioffi AM, Sinatra R, Saberski LR, Silverman DG (1991). Metoclopramide: an analgesic adjunct to patient-controlled analgesia. Anesth Analg.

[17] Rosenblatt WH, Cioffi AM, Sinatra R, Silverman DG (1992). Metoclopramide-enhanced analgesia for prostaglandin-induced termination of pregnancy. Anesth Analg.

[18] Orbach-Zinger S, Paul-Keslin L, Nichinson E, Chinchuck A, Nitke S, Eidelman LA (2012). Tramadol-metoclopramide or remifentanil for patient-controlled analgesia during second trimester abortion: a double-blinded, randomized controlled trial. J Clin Anesth.

[19] Grond S, Sablotzki A (2004). Clinical pharmacology of tramadol. Clin Pharmacokinet.

[20] Airaksinen O, Brox JI, Cedraschi C, Hildebrandt J, Klaber-Moffett J, Kovacs F (2006). Chapter 4. European guidelines for the management of chronic nonspecific low back pain. Eur Spine J.

[21] Hochberg MC, Altman RD, April KT, Benkhalti M, Guyatt G, McGowan J (2012). American College of Rheumatology 2012 recommendations for the use of nonpharmacologic and pharmacologic therapies in osteoarthritis of the hand, hip, and knee. Arthritis Care Res.

[22] Schug SA (2007). The role of tramadol in current treatment strategies for musculoskeletal pain. Ther Clin Risk Manag.

[23] Radbruch L, Grond S, Lehmann KA (1996). A risk-benefit assessment of tramadol in the management of pain. Drug Saf.

[24] Jianjing L, Yun Y (2003). Patient controlled intravenous analgesia with tramadol for labor pain relief. Chin Med J.

[25] Viegas OA, Khaw B, Ratnam SS (1993). Tramadol in labour pain in primiparous patients. A prospective comparative clinical trial. Eur J Obstet Gynecol Reprod Biol.

[26] Khazin V, Weitzman S, Rozenzvit-Podles E, Ezri T, Debby A, Golan A (2011). Postoperative analgesia with tramadol and indomethacin for diagnostic curettage and early termination of pregnancy. Int J Obstet Anesth.

[27] Karabayirli S, Ayrim AA, Muslu B (2012). Comparison of the analgesic effects of oral tramadol and naproxen sodium on pain relief during IUD insertion. J Minim Invasive Gynecol.

[28] Freidlin B, Korn EL, Gray R, Martin A (2008). Multi-arm clinical trials of new agents: some design considerations. Clin Cancer Res.

[29] Jensen MP, Miller L, Fisher LD (1998). Assessment of pain during medical procedures: a comparison of three scales. Clin J Pain.

[30] Kelly AM (2001). The minimum clinically significant difference in visual analogue scale pain score does not differ with severity of pain. Emerg Med J.

[31] Todd KH, Funk JP (1996). The minimum clinically important difference in physician-assigned visual analog pain scores. Acad Emerg Med.

[32] Schulz KF, Altman DG, Moher D (2010). CONSORT 2010 statement: updated guidelines for reporting parallel group randomised trials. BMC Med.

[33] Institute SAS (2015). The SAS system for Windows. Release 9.4.

[34] Avraham S, Gat I, Duvdevani NR, Haas J, Frenkel Y, Seidman DS (2012). Pre-emptive effect of ibuprofen versus placebo on pain relief and success rates of medical abortion: a double-blind, randomized, controlled study. Fertil Steril.

[35] Creinin MD, Shulman T (1997). Effect of nonsteroidal anti-inflammatory drugs on the action of misoprostol in a regimen for early abortion. Contraception.

[36] Lexicomp Online PaNL-DO. Hudson: Lexi-Comp, Inc.; [cited 2013 September 21]. http://www.wolterskluwercdi.com/facts-comparisons-online/.

[37] Rao AS, Camilleri M (2010). Review article: metoclopramide and tardive dyskinesia. Aliment Pharmacol Ther.

[38] World Health Organization (2002). Handbook for Good Clinical Practice (GCP): Guidance for implementation.

[39] Campbell CM, Edwards RR (2012). Ethnic differences in pain and pain management. Pain Manag.

[40] Tang OS, Gemzell-Danielsson K, Ho PC (2007). Misoprostol: pharmacokinetic profiles, effects on the uterus and side-effects. Int J Gynaecol Obstet.

[41] World Health Organization. WHO model list of essential medicines: 17th list, March 2011. 2011.

